# Evolution and comparative analysis of the MHC Class III inflammatory region

**DOI:** 10.1186/1471-2164-7-281

**Published:** 2006-11-02

**Authors:** Janine E Deakin, Anthony T Papenfuss, Katherine Belov, Joseph GR Cross, Penny Coggill, Sophie Palmer, Sarah Sims, Terence P Speed, Stephan Beck, Jennifer A Marshall Graves

**Affiliations:** 1 ARC Centre for Kangaroo Genomics, Research School of Biological Sciences, The Australian National University, Canberra, ACT 0200, Australia; 2 Bioinformatics Division, The Walter and Eliza Hall Institute of Medical Research, 1G Royal Parade, Parkville, Victoria 3050, Australia; 3 Centre for Advanced Technologies in Animal Genetics and Reproduction, Faculty of Veterinary Science, The University of Sydney, NSW 2006, Australia; 4 Wellcome Trust Sanger Institute, Genome Campus, Hinxton, Cambridge CB10 1SA, UK

## Abstract

**Background:**

The Major Histocompatibility Complex (MHC) is essential for immune function. Historically, it has been subdivided into three regions (Class I, II, and III), but a cluster of functionally related genes within the Class III region has also been referred to as the Class IV region or "inflammatory region". This group of genes is involved in the inflammatory response, and includes members of the tumour necrosis family. Here we report the sequencing, annotation and comparative analysis of a tammar wallaby BAC containing the inflammatory region. We also discuss the extent of sequence conservation across the entire region and identify elements conserved in evolution.

**Results:**

Fourteen Class III genes from the tammar wallaby inflammatory region were characterised and compared to their orthologues in other vertebrates. The organisation and sequence of genes in the inflammatory region of both the wallaby and South American opossum are highly conserved compared to known genes from eutherian ("placental") mammals. Some minor differences separate the two marsupial species. Eight genes within the inflammatory region have remained tightly clustered for at least 360 million years, predating the divergence of the amphibian lineage. Analysis of sequence conservation identified 354 elements that are conserved. These range in size from 7 to 431 bases and cover 15.6% of the inflammatory region, representing approximately a 4-fold increase compared to the average for vertebrate genomes. About 5.5% of this conserved sequence is marsupial-specific, including three cases of marsupial-specific repeats. Highly Conserved Elements were also characterised.

**Conclusion:**

Using comparative analysis, we show that a cluster of MHC genes involved in inflammation, including *TNF, LTA *(or its putative teleost homolog *TNF-N*), *APOM*, and *BAT3 *have remained together for over 450 million years, predating the divergence of mammals from fish. The observed enrichment in conserved sequences within the inflammatory region suggests conservation at the transcriptional regulatory level, in addition to the functional level.

## Background

The Major Histocompatibility Complex (MHC) is critical to the immune response of jawed vertebrates. It contains genes essential to both the adaptive and innate immune systems. Genes within the MHC have been traditionally divided into three different subregions. Class I and II regions contain genes of related structure and function, and encode molecules that are responsible for antigen presentation to T cells. The Class III region genes are more heterogeneous and their definition as Class III is based on their location between Classes I and II in eutherian (placental) mammals rather than functional commonality [1].

The human Class III region, spanning approximately 700 kb, contains 61 genes and is the most gene-dense region of the human genome [2]. Class III gene content has been shown to be well conserved, with an amphibian (*Xenopus tropicalis*) [3] and a marsupial (*Monodelphis domestica*) [4], sharing most of the genes making up the human Class III region. In stark contrast, the Class III region in the chicken and quail is represented by only a single gene, coding for a complement component gene (*C4*)[5,6]. Teleost Class III organisation is different again, with the MHC split over several different chromosomes. A survey of the *Fugu rubripes *genome for human Class III orthologues uncovered Class III genes on 31 different scaffolds [7]. A similar survey in zebrafish found that, although there is a cluster of Class III genes on chromosome 19, Class III loci are spread across all chromosomes [8].

Seven genes within the human Class III region, from *MIC *to *SKI2W *and including the tumour necrosis factor family, are thought to be involved in the inflammatory response. These genes have been referred to as the Class IV region or the inflammatory region [9]. Linkage analyses have revealed associations of the inflammatory region with many diseases, although in most cases the causal genes have remained unidentified. For instance, a region between *NFKBIL *and *MICA *may control susceptibility to hepatitis C virus-associated dilated cardiomyopathy, but the precise gene or sequence involved in this disease susceptibility remains unknown [10].

If gene content and arrangement within the inflammatory region is important for function, we would expect that the same genes would be clustered in other species as well. The Class III region is indeed highly conserved, but a comparison of the mouse and human inflammatory regions reveals that not all genes are represented in both species. The mouse inflammatory region has no functional copy of *NCR3*, and lacks both *MIC *and *MCCD1*. Pairwise alignment of the human and mouse Class III regions identified conserved non-coding elements which may act as regulatory elements, and even detected the presence of previously unrecognised genes [2].

It has been shown that the power of comparative sequence analysis can be greatly increased by the inclusion of one or more non-eutherian mammals [11]. Marsupials diverged from eutherian ("placental") mammals about 180 million years ago (MYA) [12] and neatly fill the phylogenetic gap between the bird-mammal divergence (310MYA) and the eutherian radiation (100MYA). Their genomic sequences are more easily aligned to human than are those of birds or fish but, in general, have also diverged enough for important conserved elements to be easily detected. Sequences that are highly conserved between eutherians and marsupials are likely to be functionally important, as non-functional sequences would have diverged over such a long period of evolutionary time [13].

Recent characterisation of the MHC of the grey short-tailed opossum (*M. domestica*) demonstrated that the eutherian MHC organisation is derived, whereas marsupials retain an organisation resembling the ancestral vertebrate arrangement in which the Class III region adjoins a combined Class I and II region [4]. Although the gene content and order in the opossum Class III region is well conserved compared to eutherians, there are some differences, such as the absence of two genes (*NCR3 *and *LST1*) from the inflammatory region. The Australian model marsupial, the tammar wallaby (*Macropus eugenii*), last shared a common ancestor with the opossum about 70 million years ago [14], providing a comparison similar to that of human/mouse. Given the differences between the human and mouse MHCs, comparison of gene content and arrangement in a second marsupial species will permit identification of lineage-specific changes, and identify potentially important conserved non-coding sequences.

To date, comparative studies of the Class III region have focused on the conservation of gene content and arrangement. The only large scale sequence-based comparison of the region is between human and mouse [2]. The availability of Class III sequences from other eutherian and marsupial species permits a multiple species analysis to be performed, adding power to the comparative analysis. Moreover, recent advances in the identification of conserved elements incorporate the phylogenetic relationship between species [15-18] thereby reducing bias resulting from the use of closely related species.

Here we report the characterisation of a BAC harbouring wallaby inflammatory region genes, and extend the comparative analyses of this region over a wider phylogenetic distance. Our wallaby data has allowed us to chart the evolution of the inflammatory region gene content and organisation, to perform in-depth analysis of conserved mammalian non-coding and coding sequences and to identify marsupial-specific conserved sequence elements. Identification of such elements will assist in correlating disease-associated polymorphisms with sequences of functional importance [2], assist in the identification of conserved transcription factor binding sites and ultimately result in a better understanding of gene function within the inflammatory region.

## Results

### Comparative analysis of gene content and arrangement of the inflammatory region

**Figure 1:**
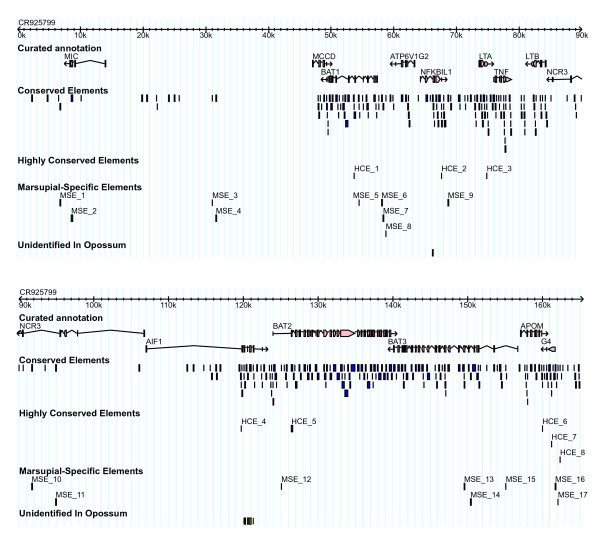
**Gene map and predicted conserved elements**. The gene annotation is based on curated GenomeScan predictions and known tammar wallaby sequences (*TNF *and *LTA*). Conserved Elements were predicted using phastCons [15] and three subsets – the Highly Conserved Elements, Marsupial-Specific Elements and Unidentified In Opossum – were identified from the percent identity between the reference sequence and each of the aligned species. This data can be viewed on the tammar wallaby inflammatory region browser [21].

We sequenced a 165 kb BAC containing a portion of the tammar wallaby MHC Class III region [EMBL: CR925799]. The orthologues of 14 human Class III genes were identified using a combination of TBLASTN and GenomeScan (Figure 1). Predicted genes were hand-curated using orthologous sequences as guides. The sequence identity of the predicted proteins are shown in Table 1. We do not have a complete sequence for *MIC*, as the 3' end of the gene is beyond the boundaries of the BAC.

Orthologues of the 14 wallaby genes were examined in other species. Gene content, structure, order and transcriptional orientation within this region are well conserved between the two marsupials (wallaby and opossum) with one noticeable exception: the absence of *NCR3 *in the opossum. A search of the opossum genome assembly (MonDom4) failed to detect a copy of *NCR3 *elsewhere in the genome. The presence of the opossum orthologue of *AIF1 *is yet to be confirmed. Only the first and part of the last exon of *AIF1 *were detected in the opossum genome assembly (MonDom4), but since these exons flank an unsequenced gap in the assembly, we assume that *AIF1 *is present.

**Figure 2:**
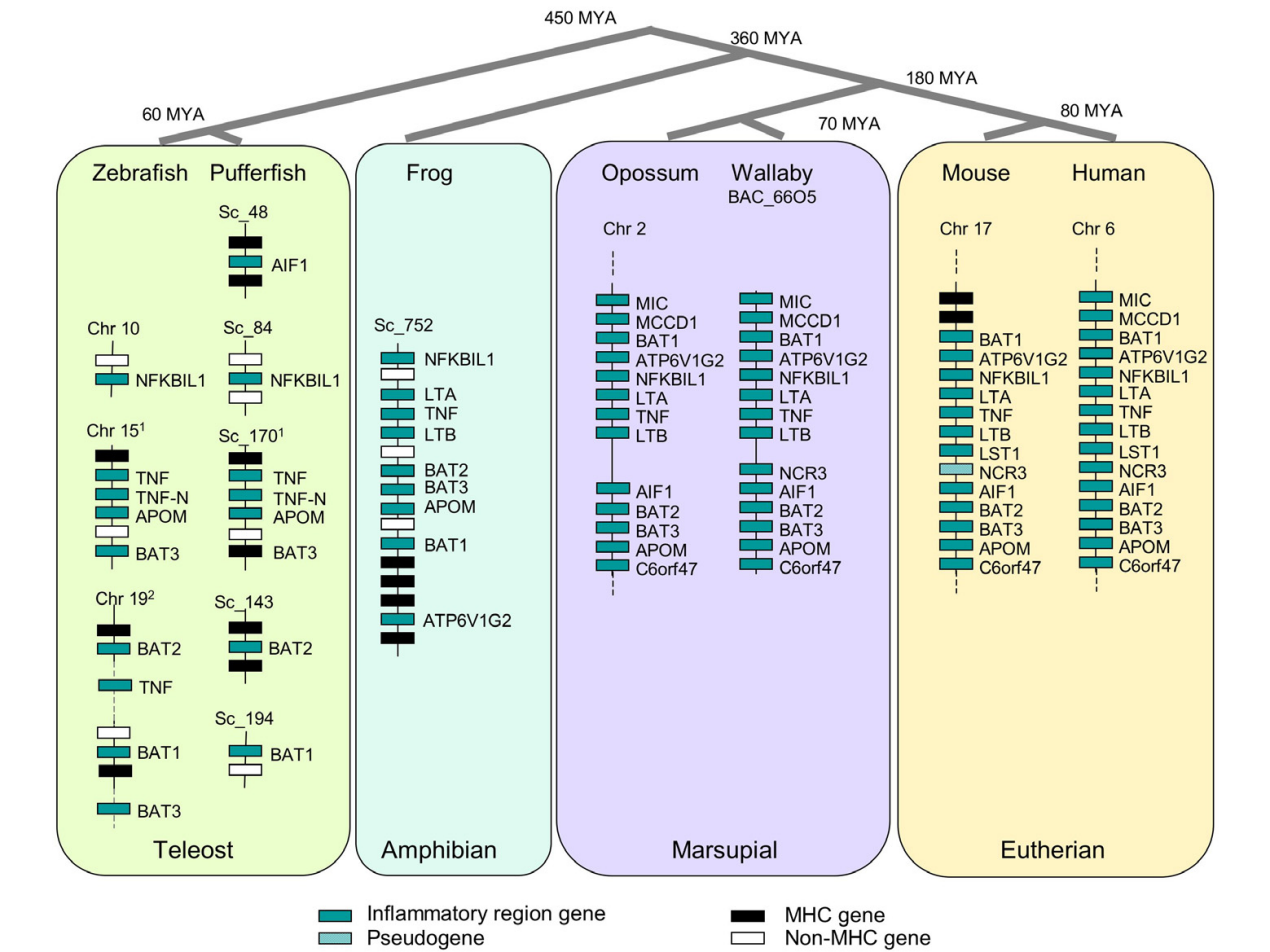
**Organisation of the inflammatory region genes across vertebrates**. Class III gene orthologues were obtained from the Ensembl genome databases [60] for mouse (*Mus musculus*) (NCBI m36), opossum (*M. domestica*) (MonDom4), frog (*X. tropicalis*) (JGI 4.1), pufferfish (*Takifugu rubripes*) (FUGU 4.0) and zebrafish (*Danio rerio*) (Zv 6). Gene organisation was determined for each species based on these genome assemblies as well as from previously published work for opossum [4] zebrafish [8, 61] and frog [3].

We next determined the organisation of genes across the interval sequenced in the wallaby (Figure 2). For the mammalian species, the genes form part of the MHC cluster and their order is conserved. Twelve of the predicted wallaby genes have the same number of exons as their human counterparts. Wallaby *NFKBIL1 *is predicted to have one fewer exon than the human orthologue.

A comparison of marsupial and eutherian inflammatory regions reveals that one gene, *LST1*, is missing from this region in the marsupials. *LST1 *sequence similarity between eutherian species is low (only 36% similarity between human and mouse), so the marsupial orthologue may have diverged to such an extent that it is difficult to detect. However, a HMMER [19] search using the *LST1 *profile from PFAM [20] reveals no significant hits and GenScan predicts no additional genes in this BAC. At this stage, we cannot exclude the existence of *LST1 *in another region of the genome, though we think this is unlikely, as no *LST1 *orthologue was detected within the MHC or elsewhere in the opossum genome.

Chicken orthologues of the MHC Class III region genes were not identified. Orthologues of several genes were found in frog (*BAT1, ATP6V1G2, NKFBIL1, LTA, TNF, LTB, BAT2, BAT3, APOM*) and were located on the same genomic scaffold, but no orthologues of five genes (*MIC, MCCD1, NCR3, AIF1 *and *C6orf47*) were detected anywhere in the frog genome. In the teleosts, orthologues for eight genes (*BAT1, NKFBIL1, LTA, TNF, LTB, BAT2, BAT3*) were distributed over several chromosomes in zebrafish (*Danio rerio*) or scaffolds in pufferfish (*Takifugu rubripes*). *AIF1 *was identified in pufferfish but not zebrafish. Genes were not grouped together in the same combinations in fish, but many were found with other genes from the eutherian Class III region. *TNF, LTA *or *TNF-N*, *APOM *and *BAT3 *were found on the same chromosomal segment in all species studied (excluding chicken) and in a similar transcriptional orientation.

### Repeat analysis

Analysis of the distribution of repeats in the wallaby BAC compared with the orthologous region in opossum and eutherians is shown in Table 2. Wallaby and opossum have similar percentages of LINEs (Long Interspersed Elements), SINEs (Short Interspersed Elements) and DNA elements, but the opossum has a much higher percentage of LTRs (Long Terminal Repeats). Most of these LTRs are endogenous retroviral elements, located mainly between *MIC *and *MCCD1 *and *LTB *and *BAT2*. This analysis also revealed a much lower percentage of SINEs in marsupials compared to eutherians whereas marsupials have a much higher percentage of LINEs than eutherians.

### Sequence conservation

We analysed the sequence conservation in wallaby- and human-referenced alignments of the MHC Class III inflammatory regions from eight mammalian species (human, chimp, mouse, rat, dog, cow, opossum and wallaby) using a phylogenetic hidden Markov model (phylo-HMM). There were 354 conserved elements predicted in the wallaby inflammatory region, covering 15.6% of the sequence (25,834 bp). Element lengths ranged from 7 to 431 bases, with a median length of 43 bases [see Additional file 1]. Predictions from both referenced alignments are compared in Table 3. The locations of conserved elements may be viewed using Gbrowse [21].

**Figure 3:**
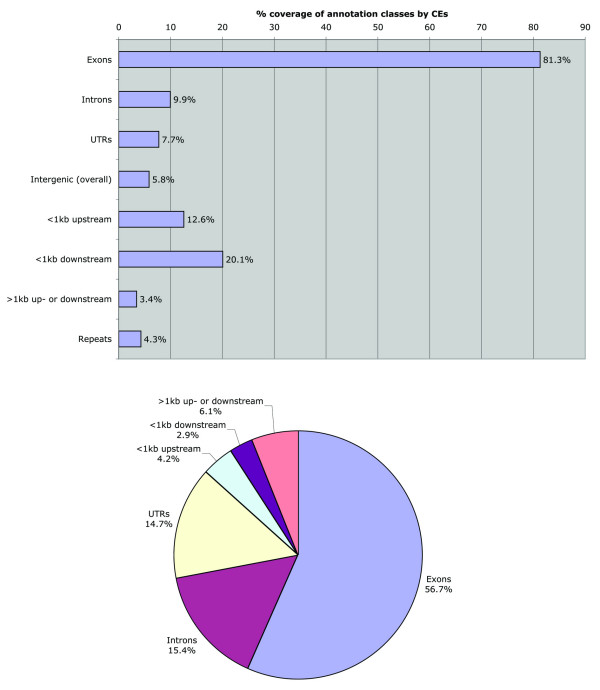
**Characterisation of conserved elements in the tammar wallaby and human inflammatory regions**. The proportion of bases in exons, introns, untranslated regions (UTRs), repeats, and intergenic sequence that are covered by conserved elements (top) and the proportion of conserved bases in each annotation class (bottom) for the wallaby-referenced sequence alignment.

To investigate the distribution of conserved elements across various sequence types, we defined seven annotation classes: exonic, intronic, untranslated regions (UTRs), repeats, and intergenic, which we further broke down into less than 1 kb upstream of an annotated gene, less than 1 kb downstream of an annotated gene, and more than 1 kb from an annotated gene. With the exception of *TNF *and *LTA*, for which the wallaby sequences are known, genes were predicted in wallaby using GenomeScan and checked by comparison with other species. Since GenomeScan does not predict UTRs, sequence for the UTR class was defined to extend from the predicted promoter to the translation start site, and from the translation stop to the poly-A tail. This approximation is likely to over-estimate the amount of sequence in this class and include intronic sequence that may not be conserved. For the wallaby-referenced alignment, the proportion of bases that are conserved in each sequence class and the distribution of conserved bases across classes are shown in Figure 3. We found that about 10% of non-coding sequence was conserved, and that about 29% of the predicted conserved sequence was non-coding.

### Highly conserved and lineage-specific elements

In order to identify highly conserved elements and lineage-specific conservation, we calculated the percent identity between the reference sequence and each of the aligned species within each of the conserved elements described above [see Additional file 2]. Elements with at least 90% identity between the reference and each aligned species were labelled Highly Conserved Elements (HCEs). Three types of lineage-specific element were also defined: Marsupial-Specific Elements (MSEs), Eutherian-Specific Elements (ESEs) and elements that are present in eutherians and wallaby, but lost or highly diverged in opossum, which we termed Undetected In Opossum (UIO). Table 4 provides the definitions of these elements. A summary of the properties of the HCEs and various lineage-specific elements is given in Table 5. Elements obtained from the wallaby-referenced alignment may be viewed using Gbrowse [21].

Eight HCEs were identified: two overlap the poly-A tails of *NFKBIL1 *and *APOM*, one is located in the 3'-UTR of *LTA*, one is found in the exon of *C6orf47*, another in the 5'-UTR of that gene, and there is one overlapping the first exon of *BAT2*. *AIF1 *has an HCE just upstream of its 5'-UTR. In the human genome, it overlaps a number of human and non-human ESTs e.g. human [DDBJ:AV735626], [GenBank: BF967128], mouse [GenBank: AI046587] and pig [GenBank: DN100732], and may represent additional 5'-UTR sequence. *BAT1 *contains an HCE in intron 5. It overlaps the start of the C/D box small nucleolar RNA mU83, which has previously been identified in the fifth intron of human, mouse and pig *BAT1 *[22].

Seventeen MSEs were identified from the wallaby-referenced alignment. These elements are highly conserved between wallaby and opossum, but the eutherian sequences contain many alignment gaps. About 5.5% of bases that are conserved are MSEs. MSEs tend to be long (42–146 bp), because phastCons requires a longer stretch of conserved sequence when conservation is limited to just a few closely related species in the multiple alignment. Thus, shorter MSEs may have been missed. The MSEs are distributed across the entire inflammatory region. Only three MSEs overlap repeats identified using RepeatMasker (MSE_10 contains an LINE (L1-2); MSE_11 overlaps with a SINE/MIR3; MSE_14 overlaps with a LINE (L3)). Two genes (*MIC *&*BAT3*) have exons containing MSEs. The 33 kb repeat-rich region between *MIC *and *MCCD1*, which appears to be expanded in marsupials, contains two MSEs, although they do not coincide with identified repeats. The remaining MSEs are in introns (seven elements) or in intergenic regions.

There were seven elements conserved in the wallaby and eutherians, but apparently not in the opossum. We termed these elements Unidentified In Opossum (UIOs). Six UIOs overlap exons of *AIF1 *(exons 2, 3, 4, 5 and 6), which are present in wallaby and well conserved in eutherians (80% identity between wallaby and human). This led us to examine the opossum sequence in this region more closely and revealed that there is a large sequencing gap in the genome assembly here. The other UIO is located in the second intron of *NFKBIL1*. It is a 15 bp segment of a SINE/MIR element that is well conserved between the eutherians and wallaby, but was not found in the opossum.

## Discussion

The comparison of genomes from an array of mammals, or even vertebrates, is key to identifying functionally important non-coding regions and gaining a better understanding of genome biology and evolution. Comparison of the wallaby BAC containing orthologues of the human Class III inflammatory region with the orthologous region in other vertebrates provides new insights into the evolution and regulatory organisation of the mammalian MHC inflammatory region.

### Gene conservation of region in mammals

By comparing the gene content and arrangement in the wallaby inflammatory region to the opossum and eutherians, we can reveal the evolutionary history of the region. The timing of events such as the loss or movement of genes from the inflammatory region can be ascertained. A comparison between the wallaby and opossum reveals that one gene (*NCR3*) is present in the wallaby but absent in the opossum. Comparative analysis of the marsupial and eutherian inflammatory regions reveals that *LST1 *became part of the region after the divergence of marsupials and eutherians. It is also apparent that the mouse is more divergent in gene content than the other eutherian species, lacking both *MCCD1 *and *MIC *in addition to having only a non-functional copy of *NCR3*.

Comparing the wallaby BAC sequence with the orthologous region in other mammals revealed a high level of similarity between the gene content and organisation of marsupials and eutherians. Variation in the level of conservation between the wallaby genes and other mammalian orthologues is apparent at the protein and nucleotide levels (Table 1). The most highly conserved are those loci with putative housekeeping functions. For instance, wallaby *BAT1*, a member of the DEAD box protein family of ATP-dependent RNA helicases, is 99% identical to human *BAT1 *at the amino acid level. The least conserved genes have products with specialised immune function, such as *NCR3*. This level of sequence divergence is characteristic of genes of immunological importance because they evolve rapidly to counter rapidly evolving pathogens.

The presence of *NCR3 *in wallaby but its absence in opossum is not unexpected, since *NCR *gene content and function differs also between human and mouse [23]. *NCR3 *is a member of the gene family specifying natural cytotoxicity receptors, which are responsible for natural killer (NK) cell activation [24]. In humans there are three expressed *NCR *genes but in mouse *NCR1 *is the only active gene, *NCR2 *having been lost [23] and *NCR3 *a pseudogene [2,23]. The three human *NCR*s appear to have adopted specialised functions. *NCR1 *functions as the predominant activating NK receptor, whereas *NCR2 *and *NCR3 *have much more restricted expression profiles [23]. This suggests duplication of an original *NCR *gene with the specialization of duplicate genes in humans, and their loss independently in mice and opossums. The present study implies that these duplications took place much earlier than had been supposed, before the divergence of marsupials and eutherians.

One of the most obvious differences between marsupials and eutherians is the apparent absence of an *LST1 *orthologue in both marsupial species studied. Sequence similarity between eutherian species is low for this gene so marsupial *LST1 *may have diverged to such an extent that it is difficult to detect. Nonetheless, even our more rigorous search for this gene in the opossum using HMMER failed to detect an *LST1 *orthologue in the opossum genome. The function of *LST1 *is still being elucidated but it has been shown to have an immunomodulatory function with a very strong inhibitory effect on lymphocyte proliferation [25] and its expression is up-regulated in response to bacterial infection and inflammatory mediators [26]. Splice variants result in either membrane-bound or soluble *LST1 *isoforms that presumably carry out different functions.

Mixed lymphocyte reactions (MLR) test the recognition and proliferation of T lymphocytes in culture. Marsupials have a weak or even non-existent mixed lymphocyte response yet mount a normal graft rejection response [27]. Initially, it was thought that this weak MLR was the result of a low level of MHC Class II variability [28], an amino acid substitution in the MHC molecule [27] or even a difference in the ontogeny of marsupial and eutherian T cells [29]. The absence of immunomodulation by *LST1 *may also be a contributing a factor.

Our analysis of *MCCD1 *(Mitochondrial Coiled-Coil Domain) shows that this gene is much older than had been previously thought. This gene is present in the human MHC Class III region [30], but is evidently lacking from this region in mouse; nor is there an orthologue elsewhere in the mouse genome. A previous study failed to detect *MCCD1 *orthologues in GenBank genome and EST databases for any other species other than pig and it was suggested that *MCCD1 *emerged 60 – 100 million years ago, prior to the divergence of pigs but after the divergence of rodents [30]. However, our demonstration of a *MCCD1 *orthologue in the wallaby region reveals that this gene has been present in the mammalian lineage for over 180 million years, since marsupials and eutherians last shared a common ancestor, so was probably lost in the rodent lineage.

Part of a wallaby *MIC *orthologue was identified within the BAC, lacking only the terminal exon and polyadenylation signal. This wallaby *MIC *orthologue shows sequence identity to the human *MIC *gene and the murine *Mill *genes. Phylogenetic analysis indicates the sequence is basal to both *MIC *and *Mill *(data not shown). The presence of the *MIC *orthologue in marsupials suggests that the *Mill *gene family in the mouse was derived from a *MIC *gene ancestor that has diverged, possibly as a result of having moved out of the MHC [4].

Thus, the highly conserved mammalian inflammatory region has been relatively stable over the last 180 million years, with the apparent gain of one gene (*LST1*) in eutherians. *NCR3 *has undergone lineage specific changes, having been lost from the opossum and non-functional in the mouse but the presence of this gene in the wallaby indicates that it was part of the inflammatory region prior to marsupial/eutherian divergence. The stability of this region in mammals over the past 180 million years suggests that the maintenance of these genes in a cluster may be functionally important.

### Evolution of the MHC Class III region

Comparison of the inflammatory region of the wallaby MHC Class III with that of other vertebrates indicates that extensive evolutionary changes occurred early in vertebrate evolution, but were followed by a lengthy period of evolutionary stability. We have shown that many of the genes making up the inflammatory region of the eutherian MHC have been clustered for at least 350 million years. Frog *NFKBIL1, LTA, TNF, LTB *and *BAT2, BAT3, APOM *are in the same transcriptional orientation as in mammals, although there have been several rearrangements in this region. The gene *RSG3*, located between *LTB *and *BAT2*, moved to a different chromosome and the *LST1 *gene was inserted in eutherians. *NCR3 *and *AIF1 *appear to have been lost in some lineages. *BAT1 *and *ATPV1G2 *have moved and changed transcriptional orientation.

The three genes *TNF, LTA *and *LTB *of the eutherian tumour necrosis factor family genes are arranged in tandem, with *TNF *and *LTA *in the same transcriptional orientation and *LTB *in the opposite orientation. This formation is conserved, even in marsupials, suggesting that there may be some functional advantage to this arrangement. The proteins encoded by *TNF, LTA *and *LTB *are known to interact as complementary factors in various cell signalling networks [31,32]. Hence, the maintenance of this gene arrangement may serve to facilitate co-ordinated regulation of these genes.

The absence of genes from some lineages does not necessarily mean that the functions of these genes are lost. For instance, chicken lacks *TNF*, but the its paralogue (*TL1A*) appears to function as a *TNF *substitute [33]. Likewise, in the lineages that lack the allograft inflammatory factor *AIF1*, a paralogue may have assumed its function. *AIF1 *is a cytokine-responsive, calcium binding protein, shown to play a role in allograft rejection [34,35] and in the inflammatory response following injury [36]. An orthologue of this gene was identified in eutherians, marsupials and pufferfish but not chicken, frog or zebrafish. A paralogue of this gene (*C9orf58*) is found on human chromosome 9 and is also present in chicken and frog, flanked by other human chromosome 9 genes. This gene may take over the role of *AIF1 *in lineages where *AIF1 *has been lost.

Our analyses suggest that eight genes within the inflammatory region have remained tightly clustered for at least 350 million years, predating the divergence of the amphibian lineage. The linkage of *TNF, LTA *(or its putative teleost homolog *TNF-N*), *APOM*, and *BAT3*, predates the divergence of teleosts and mammals some 450 million years ago and is consistent with a functional significance of clustering of genes involved in inflammation. Chicken is the exception, with no inflammatory region orthologues identified but it is likely that paralogues of these genes perform their function.

### Sequence conservation

Genome comparisons between evolutionary diverse species have proven particularly useful for detecting conserved sequences, which may be functionally important. Older pairwise methods for detecting sequence conservation (e.g. VISTA and PipMaker [37,38]) required the careful selection of species to avoid biasing predictions by including closely related species (e.g. human, mouse, rat). More recent methods take the phylogenetic relationship of species into account to reduce or eliminate this bias (e.g [15,17,18]). However, when using these methods, careful species selection is still important. As with pairwise methods, there is a trade-off between alignability and evolutionary depth. Also, including too many species that lack a gene or region of interest will reduce the sensitivity of conserved element prediction.

Marsupials are extremely well placed phylogenetically for the analysis of the inflammatory region, which is highly conserved amongst eutherians, but completely absent from the chicken genome. We found that the frog inflammatory region, while reasonably well conserved in terms of gene content, does not align well to the mammalian inflammatory region in non-coding regions. In teleosts, the inflammatory region is split over different chromosomes and obtaining a high quality alignment outside of coding regions is extremely difficult.

We searched the highly gene dense MHC Class III region for conserved elements using phastCons, an approach that utilizes a phylogenetic hidden Markov model. We identified 354 conserved elements covering 25,834 bp or 15.6% of the region. Siepel et al. [15] used this approach to identify conserved elements in genomes of five vertebrates (human, mouse, rat, chicken and fugu). They found 1.18 million conserved elements covering 4.3% of the human genome. Thus we find roughly an eight-fold enrichment in the number of conserved elements in the inflammatory region and a four-fold increase in the proportion of sequence covered compared to a uniform distribution at this rate. The high density of conserved elements in this gene-rich region itself suggests that they have a function in regulation of the genes.

The UCSC Genome Browser "Most Conserved" track for the human genome (hg17, March 2006) is based on a phastCons analysis of a human-referenced multiz alignment of 17 vertebrate species (human, chimpanzee, rhesus, mouse, rat, rabbit, dog, cow, armadillo, elephant, tenrec, opossum, chicken, frog, zebrafish, and the two pufferfish species *Tetraodon nigroviridis *and *Takifugu rubripes*). It identifies 302 conserved elements, covering 23470 bp, in the human inflammatory region. This is substantially less than the 468 elements, covering 30,192 bp that we identified in human. Examining the 17-way alignment of the inflammatory region reveals that only human, mouse, rat, dog and opossum are aligned substantially, with regions from chicken, *X. tropicalis *and *T. nigroviridis *aligning in smaller segments. Thus, locally the sequence alignment contains fewer eutherians, only one marsupial and many sequence gaps from the non-mammals. It is likely that the deeper phylogenetic coverage and fewer mammalian sequences (particularly the absence of cow and wallaby) make the UCSC phastCons analysis less sensitive for small mammal-specific conserved elements. This demonstrates that, while the UCSC genome browser is an excellent resource for comparative genomics, there is still some role for careful selection of species, taking the biology into account, when analysing sequence conservation.

The proportion of exonic and intronic sequence in the inflammatory region that is covered by conserved elements is much higher (81% and 10% respectively) than the genomic averages (66% and 3.6% respectively) [16], reflecting the high conservation of the inflammatory region. This is true of all annotation classes except the UTR class, which is poorly resolved, and contains a lower proportion of conserved bases because the amount of sequence in this class was over-estimated. Due to the high conservation together with the high gene density of this region, a much larger proportion of conserved elements occur in exons (57%) and a much lower proportion in intragenic regions (13%) than the genomic averages (18% and 41.2% respectively).

We calculated the pairwise similarity of sequences within phastCons-predicted conserved sequences. This simple *post hoc *analysis lacks the sophistication employed in the original prediction, but was effective in stratifying conserved elements into different classes and revealing clade-specific features in the eutherian and marsupial lineages. These elements are likely to include known and novel repeats, sequence retro-transposed into the region after the divergence of eutherians and marsupials, clade-specific features in proteins and clade-specific regulatory regions. Such clade-specific elements could be evolutionarily important, as changes in gene regulation, rather than gene products, are likely to play a major role in the phenotypic differences between marsupials and eutherians [13]. New methods are now emerging that incorporate the phylogeny into the prediction of conserved elements and identify lineage-specific elements [39].

### Conserved regulatory elements

It is important to categorize conserved elements based on their different patterns of molecular evolution to gain insight into the potential function of the element [13]. There are 28 conserved elements within 1 kb upstream and 17 within a 1 kb of the 3' end of any annotated gene. Whereas regulatory regions can occur further up- and downstream from genes, as well as within introns, these closer elements are the best candidates for transcription factor binding sites and regulatory modules. Indeed, the previously identified conserved element upstream of *TNF *(-207 to +27) with 65–70% sequence identity between wallaby and eutherian sequences and 86% identity between wallaby and opossum [43], contains the known human and mouse regulatory module of *TNF *consisting of three *EST-1/ELK-1*, a *Jun-ATF2*, three *NFAT *and a *Sp-1 *binding site [31,32].

*TNF *is a cytokine involved in the regulation of a diverse spectrum of biological processes such as cell proliferation, lipid metabolism, apoptosis, and coagulation. An excess of *TNF *results in harmful inflammatory responses [40], whereas under-expression for *TNF *has been shown to contribute to pathogen susceptibility [41]. *TNF *has been implicated in an array of diseases including autoimmune disease and cancer. A number of single nucleotide polymorphisms (SNPs) within the promoter have disease associations. A SNP at position -572 is associated with a susceptibility to papillovirus 16-associated cervical cancer [42]. The conserved element we identified encompassing this -572 polymorphic site may therefore be important to the regulation of *TNF *transcription. Many other SNPs have been reported in the promoter region of *TNF*, some with disease associations, but there are conflicting reports in the literature on whether these polymorphisms have a significant effect of *TNF *expression [43]. Recognition of conserved elements will assist in identifying SNPs in the *TNF *promoter that are likely to be functionally important.

Disease associated SNPs are not limited to protein coding sequence or upstream elements but can also be found within introns. A SNP within intron 1 of human *LTA *enhances the transcriptional level of this gene, indicating that it is important for its transcriptional regulation [44]. Individuals with this allele have been shown to have an increased susceptibility to myocardial infarction as well as large-vessel-associated ischemic stroke [45]. As more disease association studies are carried out on the inflammatory region genes, it becomes increasingly important to identify sequences which are potentially important to gene function and regulation so that functional disease associations can be detected. By cataloguing conserved elements within this region it should be easier to correlate SNPs with functionally important sequences.

## Conclusion

We have constructed a rich dataset of mammalian sequence from the well-conserved MHC Class III inflammatory region and used comparative methods to study its evolution and analyse its sequence conservation. Our results confirm the power gained by using two distantly related marsupial species in comparative studies.

The wallaby genes in this region are highly conserved in organisation and sequence relative to known eutherian sequences. Analyses across taxa show that the linkage of *TNF, LTA *(or its putative teleost homologue *TNF-N*), *APOM *and *BAT3 *predates the divergence of teleosts and mammals some 450 million years ago, consistent with the functional importance of the clustering of MHC genes.

We have examined the character of sequence conservation in the mammalian inflammatory region and identified clade-specific elements. This is a necessary first step towards identifying new regulatory elements, and may prove useful for linking SNPs associated with disease susceptibility to gene function.

## Methods

### BAC sequencing

BAC MeVIA_66O5 was previously isolated, confirmed to contain *LTA, TNF *and *LTB *[46] and selected for complete sequencing. For the shotgun phase [47], pUC plasmids with inserts of mostly 1.4–2 kb were sequenced from both ends using the dideoxy big dye terminator chemistry [48]. The resulting sequencing reactions were analysed on ABI sequencing machines and the generated data were processed by a suite of in-house programs [49] prior to assembly with the PHRED [50,51] and PHRAP [52] algorithms. For the finishing phase, we used the GAP4 program [53] to help assess, edit and select reactions to eliminate ambiguities and close sequence gaps. Sequence gaps were closed by a combination of primer walking, PCR, short/long insert sublibraries [54], oligo screens of such sublibraries and transposon sublibraries. The finished sequence of MeVIA_66O5 has been submitted to the Genbank/EMBL/DDBJ databases [EMBL: CR925799].

### Sequence annotation

Similarity features in the wallaby BAC sequence were found by aligning proteins from the human RefSeq collection with the wallaby sequence using TBLASTN[55]. Three known tammar wallaby genes from the region, *TNF *[GenBank: AF055915], *LTA *[GenBank: AF119336] and *LTB *[GenBank: AY853666] were aligned with the wallaby BAC sequence using BLAT [56]. Gene prediction was performed using GenomeScan [57], which was supplied with human RefSeq proteins from the MHC region as its putative homolog set. Repeats were identified using RepeatMasker [58]. The annotation was then visualized using GBrowse [59]. A curated annotation was constructed by integrating the various annotation tracks and hand annotating individual genes.

Wallaby open reading frames and protein sequences were predicted based on the orthologous human sequence *MCCD1 *[RefSeq:XP_376486]; *BAT1 *[RefSeq:NP_004631]; *ATP6V1G2 *[RefSeq: NP_569730]; *NFKBIL1 *[RefSeq:NP_004998]; *TNF *[RefSeq:NP_000585]; *LTB *[RefSeq:NP_002332]; *NCR3 *[RefSeq:NP_667341]; *AIF1 *[RefSeq:NP_001614]; *BAT2 *[RefSeq:NP_542417]; *BAT3 *[RefSeq:NP_004630;] *APOM *[RefSeq:NP_061974]; *C6orf 47 *[RefSeq:NP_067007]. Class III gene orthologues were obtained from the Ensembl genome databases [60] for mouse (*Mus musculus*) (NCBI m36), dog (*Canis familiaris*) (CanFam1.0), opossum (*M. domestica*) (MonDom4), frog (*X. tropicalis*) (JGI 4.1), pufferfish (*Takifugu rubripes*) (FUGU 4.0) and zebrafish (*Danio rerio*) (Zv 6) as well as from previously published work on zebrafish [8,61] and frog [3].

### Analysis of sequence conservation

Genomic segments corresponding to the portion of the wallaby inflammatory region contained in the BAC (from *MIC *to *C6orf47 *plus 4–8 kb at either end) from the human [NCBI Build 36.1:chr6:31563944-31746528], chimpanzee [PanTro2, Oct. 2005: chr6:31950000-32137000], mouse [Mm8, February 2006:chr17:32821777–32976813], rat [Version 3.4, November 2004:chr20:3599000–3761000], and dog [CanFam2, May 2005:chr12:3930000–4090000] genomes and from the unpublished cow genome [Btau_2, March 2005:chr23:23180000–23322000] [62] were obtained from the UCSC Genome Browser [63]. The cow genome assembly was incomplete at the time this sequence was extracted, however, it seems unlikely that this would affect this region or our conclusions. Sequence from the opossum inflammatory region was obtained from the unpublished opossum genome [64] [MonDom3:scaffold_42:17788098–18043098] via the *Monodelphis *MHC browser [65]. A multiple alignment of these sequences together with the wallaby BAC sequence was constructed using MAVID 2.0 [66]. This was converted into a wallaby- and a human-referenced alignment by removing columns from the multiple alignment, where the reference sequence contained gaps.

Conserved elements were predicted from both referenced alignments using a phylogenetic hidden Markov model (HMM) consisting of two states (conserved and non-conserved) implemented in phastCons [15]. Referenced alignments were used because phastCons performed poorly on the full multiple alignment. The HMM transition probabilities were treated as tuning parameters and a variety of values, including the maximum likelihood estimates, were explored. The final analysis used μ = 0.0833 and ν = 0.03. This is the same choice used for the set of aligned vertebrate genome sequences in Siepel et al. [15]. They obtained these values by constraining the proportion of coding sequence across the human genome that was covered by conserved elements to be 65% and by imposing certain smoothness requirements on the conservation. This choice of μ also corresponds to a prior on the expected conserved element size of 12 bases. Phylogenetic models for the conserved and non-conserved state were fitted to the referenced alignment using unsupervised learning. A conservation score, the posterior probability of being in the conserved state, was also calculated. The results of conservation analysis of the wallaby-referenced sequence were visualised and integrated with annotation data in GBrowse.

## Authors' contributions

JED and ATP carried out analysis and drafted the manuscript. PC, SP and SS sequenced the wallaby BAC. TPS, SB and JAMG contributed towards establishing the framework for this study and they, as well as KB and JGRC helped in the preparation of the manuscript. All authors have read and approved the final manuscript.

## Supplementary Material

Additional File 1 **Wallaby-referenced conserved elements**. Text file containing tammar wallaby conserved element locations and sequence data. Each conserved element has a header line, starting with a '>', providing a name and location for the element. The alignment in the conserved element follows in a clustalw-like format. Click here for file

Additional File 2 **Percent identities of conserved elements**. Excel file containing percent identities of conserved elements between each reference species and the aligned sequences. Click here for file

## Figures and Tables

**Table 1:** Percent identity between wallaby proteins and orthologues

**Predicted wallaby orthologue**	**Human % identity**	**Opossum % identity**	**No. of exons in wallaby (human)**	**No. of amino acid residues in wallaby (human)**
*MCCD1*	67	87	2 (2)	115 (119)
*BAT1*	99	97	10 (10)	428 (428)
*ATP6V1G2*	95	94	3 (3)	118 (118)
*NFKBIL1*	62	77	3(4)	358 (381)
*LTA*	60	84	3(3)	201 (205)
*TNF*	70	76	4 (4)	233 (233)
*LTB*	62	78	4(4)	250 (244)
*NCR3*	39	-	4 (4)	180 (201)
AIF1*	80	N/A	6 (6)	147 (147)
*BAT2*	84	96	31 (31)	2137 (2157)
*BAT3*	84	96	25 (25)	1116 (1132)
*APOM*	71	85	6 (6)	188 (188)
*C6orf47*	56	79	1 (1)	299 (294)

Refer to Methods section for human RefSeq accession numbers. *MIC *has been excluded from this analysis due to the wallaby *MIC *sequence being incomplete.

* Only the first and part of the last exons of *AIF1 *were detected in opossum. These flank a sequencing gap in the assembly.

**Table 2:** Repeat content in the wallaby BAC and orthologous regions

**Species**	**Length (bp)**	**LINE (%)**	**SINE (%)**	**LTR elements (%)**	**DNA elements (%)**	**Total (%)**
Wallaby	165490	22.7	8.1	1.6	1.1	33.5
Opossum	255001	27.2	9.8	10.3	1.3	48.6
Human	182585	8.2	26.4	2.0	1.1	37.7
Chimp	187001	6.3	21.1	2.1	0.6	30.1
Mouse	141801	0.1	18.3	4.7	0.6	23.7
Cow	142001	14.8	10.9	2.0	1.6	29.3
Dog	160001	10.6	14.2	1.4	0.8	27.0

LINE, long interspersed element; SINE, short interspersed element; DNA elements, DNA transposons.

**Table 3:** Summary of conserved elements for each reference sequence analysed

	**Wallaby**	**Human**
Number of Conserved Elements	354	468
Median length (bp)	43	34.5
Minimum length (bp)	7	6
Maximum length (bp)	431	440
Total sequence covered (bp)	25,834	30,192
Proportion of region covered	15.6%	16.5%

**Table 4:** Definitions of highly conserved and lineage-specific elements

**Reference Species**	**Class name**	**Label**	**Definition**
Any	Highly Conserved Element	HCE	Minimum % identity > 90%*
Tammar wallaby	Marsupial-Specific Element	MSE	Average marsupial % identity < 80% Maximum eutherian % identity < 20%
Tammar wallaby	Unidentified in Opossum	UIO	Opossum % identity <10% Average eutherian % identity > 50%
Human	Eutherian-Specific Element	ESE	Average eutherian % identity > 80% Maximum marsupial % identity < 20%

*relative to reference sequence

**Table 5:** Summary of highly conserved and lineage-specific elements. The 6 categories of conserved elements are defined in Table 4.

**Reference sequence**	**Class label**	**Number of elements**	**Length range (min-max)**	**Median length**	**Total number of bases**	**Mean eutherian % identity**	**Mean marsupial % identity**
Wallaby	HCE	8	7–190	13	275	95.5	99.0
	MSE	17	42–146	89	1413	4.7	93.3
	UIO	7	15–174	44	434	80.9	0.7
Human	ESE	30	10–104	28.5	1121	88.1	2.8
